# Changes in Place of Death Among Patients With Dementia During the COVID-19 Pandemic in Japan: A Time-series Analysis

**DOI:** 10.2188/jea.JE20230279

**Published:** 2024-10-05

**Authors:** Nahoko Harada, Masahide Koda, Akifumi Eguchi, Masahiro Hashizume, Motoi Suzuki, Shuhei Nomura

**Affiliations:** 1 Department of Nursing Science, Graduate School of Interdisciplinary Science and Engineering in Health Systems, Okayama University, Okayama, Japan; 2 Co-learning Community Healthcare Re-innovation Office, Graduate School of Medicine, Dentistry and Pharmaceutical Sciences, Okayama University, Okayama, Japan; 3 Department of Sustainable Health Science, Center for Preventive Medical Sciences, Chiba University, Chiba, Japan; 4 Department of Global Health Policy, Graduate School of Medicine, The University of Tokyo, Tokyo, Japan; 5 Center for Surveillance, Immunization and Epidemiologic Research, National Institute of Infectious Diseases, Tokyo, Japan; 6 Department of Health Policy and Management, School of Medicine, Keio University, Tokyo, Japan; 7 Tokyo Foundation for Policy Research, Tokyo, Japan

**Keywords:** COVID-19, dementia, public health, aged, nursing home

## Abstract

**Background:**

A key measure of the effectiveness of end-of-life care is the place of death. The coronavirus disease 2019 (COVID-19) pandemic affected end-of-life care and the circumstances of patients with dementia.

**Methods:**

This observational, retrospective cohort study used Japanese national data to examine the numbers and locations of reported deaths among patients with dementia older than 65 years during the COVID-19 pandemic. Locations were grouped as medical institutions, nursing facilities, homes, or all settings. The quasi-Poisson regression model known as the Farrington algorithm was employed.

**Results:**

Between December 30, 2019, and January 29, 2023, 279,703 patients who died of causes related to dementia were reported in Japan. A decline was seen in early 2020, followed by increased numbers of deaths in homes, medical facilities, and nursing homes beginning in October 2020, December 2020, and March 2021, respectively. In 2021, the percentage of excess deaths at home peaked at 35.2%, while in 2022, those in medical facilities and nursing homes peaked at 18.8% and 16.6%, respectively. In 2022, the percentage of excess deaths in nursing homes exceeded that of other locations.

**Conclusion:**

The results suggest a change in the preferred place of death, along with pandemic-related visitation restrictions among healthcare facilities. Excess deaths also suggest strained medical resources and limited access to care. Methodological limitations include data from a limited period (2017 onwards) and post-2020 data used to estimate data after 2021, albeit with weighting. Considering these findings, physicians should reconfirm preferred places of death among older patients with dementia.

## INTRODUCTION

It is estimated that by 2030, 75 million individuals worldwide will be living with dementia—significantly more than the current estimate of 47 million cases.^1^ In an aging society, people of all ages require end-of-life care, and those with dementia are no exception. Whether patients are able to spend their last moments in the place of their choosing is an essential indicator of end-of-life care quality.^2^ Among people with dementia, the past two decades have witnessed a global shift from dying in hospitals to dying at home or in nursing facilities.^3^^–^^5^ While a preference for homes over nursing facilities is evident in high-income countries,^6^ a population study in Japan—another high-income country—reported that people with dementia preferred to die in nursing facilities over their homes or hospitals.^7^ However, the coronavirus disease 2019 (COVID-19) pandemic has restricted access to healthcare globally, including in Japan. The number of hospital admissions and outpatient wards decreased once the outbreak began, as did the length of stay.^8^^,^^9^ To strengthen end-of-life care systems associated with medical care and welfare services, this study explored changes in the location of death among patients with dementia during the COVID-19 pandemic in Japan, especially between January 2020 and December 2021.

## METHODS

We analyzed national mortality data from the Ministry of Health, Labour and Welfare (MHLW) of Japan from January 2017 to January 2023 to study changes in the numbers and locations of reported deaths among dementia patients during the COVID-19 pandemic. Only patients aged 65 years or older who were diagnosed with vascular dementia (International Statistical Classification of Diseases and Related Health Problems 10^th^ version^10^ [ICD-10], classification F01), Alzheimer’s disease (G30), or dementia not otherwise defined (F03) were considered. Death locations were re-categorized into four main types: all places, medical institutions, nursing facilities, and homes.^7^^,^^11^^–^^14^

For statistical analysis, the study used the Farrington algorithm, which employs the quasi-Poisson regression model.^15^^,^^16^ This method involves calculating the expected number of deaths for a given week, then comparing this number with historical data to determine excess deaths. Japan revised its primary cause of death criteria in January 2017 to align with the 2013 revision of ICD-10.^17^ Hence, data before 2017 were not used due to inconsistencies. The algorithm incorporated pre-pandemic data from 2017–2019, considering the rule change in 2017, and weighted outliers from 2020 onwards for subsequent estimates. For more detailed methodology, refer to eMaterial 1.

This study was approved by the ethical committee of the National Institute of Infectious Diseases (authorization no. 1552), and it adhered to institutional guidelines.

## RESULTS

**Figure 1.**
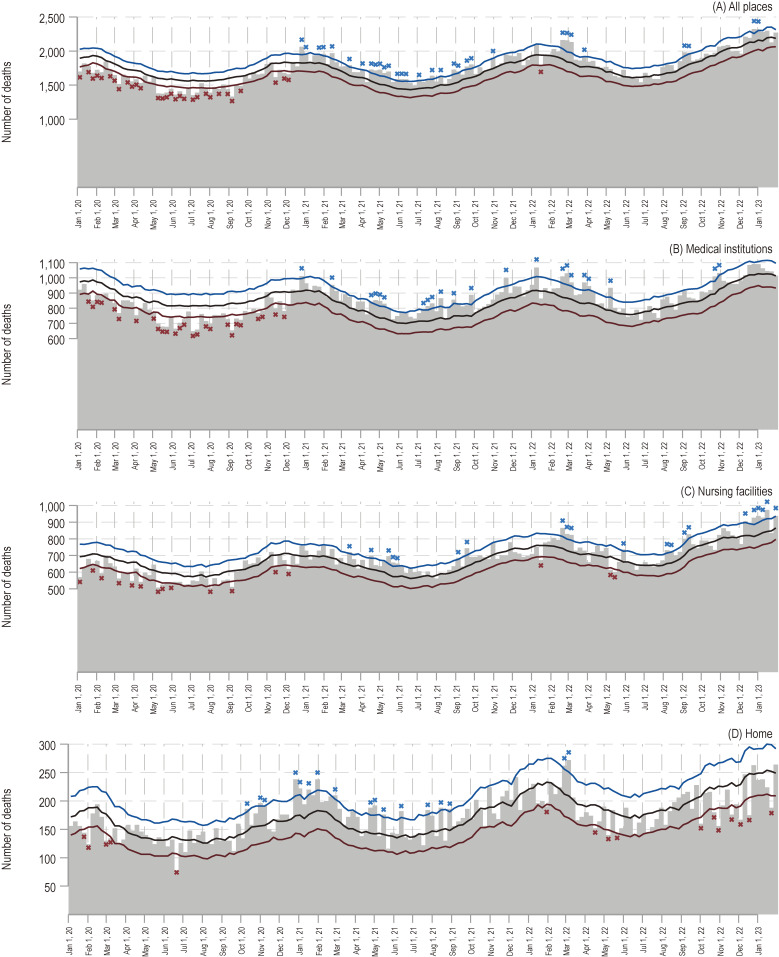
Weekly trends in the excess number of deaths among people with dementia between 2019 and 2023, categorized by place of death. This figure shows the weekly trends in the excess number of deaths between December 30, 2019, and January 29, 2023, categorized by place of death: (**A**) all places, (**B**) medical institutions, (**C**) nursing facilities, and (**D**) homes. The 95% upper and lower limits of the expected number of deaths are indicated by the blue and red lines, respectively. If the measured number of deaths per week exceeded the 95% upper limit, the number of deaths was marked with a blue cross. Conversely, if the measured number of deaths per week was below the lower 95% limit, the number of deaths is marked in red.

A total of 279,703 people with dementia died within the 160 weeks between December 30, 2019, and January 29, 2023 (Table 1 and eTable 1; abbreviations used in eTable 1 are available in eMaterial 2). A total of 136,132 (48.7%) deaths occurred in hospitals, 108,700 (38.9%) in nursing facilities, and 27,711 (9.9%) at home. The statistics of weekly expected and observed deaths, as well as the upper and lower 95% limits, are shown in eTable 1. Figure 1 illustrates the weekly trends in excess mortality numbers between December 30, 2019, and January 29, 2023, according to the place of death. The weekly trends in observed deaths between January 2015 and January 2023 are shown in eFigure 1. The weekly percent excess, a measure of the surplus’s relative size, is displayed in eFigure 2.

**Table 1.** Observed and excess deaths between 2020 and 2023, categorized by place of death

Year	2020^a^	2021^b^	2022^c^	2023^d^
**All**				
Observed, *n*	82,662	90,250	97,768	9,023
Excess, *n*	−6,115	5,287	3,115	314
Percent excess, median (IQR)	−7.4 (−10.5 to −3.9)	6.5 (4.1–9.7)	2.8 (0.6–5.7)	5.4 (2.7–6.4)
Percent excess, min, max	−17.2, 13.1	−4.0, 14.4	−8.1, 17.2	−3.5, 7.3
**Medical institutions**				
Observed, *n* (%)	41,284 (49.9)	43,956 (48.7)	46,652 (47.7)	4,020 (44.6)
Excess, *n*	−3,978	2,802	2,088	139
Percent excess, median (IQR)	−8.9 (−13.1 to −5.4)	7.1 (2.3–10.0)	3.5 (1.1–7.1)	2.8 (1.8–4.4)
Percent excess, min, max	−21.6, 11.0	−2.3, 18.9	−7.0, 18.8	1.76, 6.13
**Nursing facilities**				
Observed, *n* (%)	31,450 (38.0)	34,760 (38.5)	38,814 (39.7)	3,676 (40.7)
Excess, *n*	−1,928	1,261	1,197	323
Percent excess, median (IQR)	−6.0 (−9.9 to −1.1)	4.6 (−1.1 to 7.4)	3.1 (−1.0 to 7.2)	12.4 (7.8–14.2)
Percent excess, min, max	−17.8, 8.9	−6.2, 19.0	−12.0, 16.6	−1.4, 15.4
**Home**				
Observed, *n* (%)	7,694 (9.3)	9,232 (10.2)	9,898 (10.1)	887 (9.8)
Excess, *n*	−118	749	−690	−117
Percent excess, median (IQR)	−1.5 (−7.4 to −7.5)	8.2 (−0.8 to 19.1)	−5.7 (−14.8 to 2.1)	−8.3 (−15.2 to −4.7)
Percent excess, min, max	−42.2, 36.8	−16.6, 35.2	−31.0, 30.1	−25.4, −4.4

^a^2020: between December 30, 2019 and December 27, 2020.

^b^2021: between December 28, 2020 and December 26, 2021.

^c^2022: between December 27, 2022 and December 25, 2022.

^d^2023: between December 28 and January 29, 2023.

Figure 1A shows general trends in the weekly number of dementia-related deaths regardless of their location, together with the expected number of deaths, upper and lower 95% limits. The 1-year accumulation of weekly excess deaths calculated by subtracting the expected value from the observed value was −6,115 in 2020, 5,287 in 2021, and 3,115 in 2022. Between January and the last week of December 2020, no weeks with excess deaths, defined as a number above the 95% upper limit, were observed, while there were 30 weeks with fewer-than-expected deaths, defined as a number under the 95% lower limit. Thirty-one of the 160 weeks from the last week of December 2020 through January 2023 evidenced excess deaths. The median percent excesses were −7.4% in 2020, 6.5% in 2021, 2.8% in 2022, and 5.4% in 2023.

In medical institutions, no weeks with excess deaths were observed until December 2020, and there were fewer-than-expected deaths in 26 weeks (Figure 1B). After December 2020, there were no weeks with fewer-than-expected deaths. Twenty-two of the 160 weeks were characterized by excess deaths. The median percent excesses in 2020, 2021, 2022, and 2023 were −8.9%, 7.1%, 3.5%, and 2.8% respectively.

In nursing facilities, no excess deaths were observed until March 2021, and there were fewer-than-expected deaths in 13 weeks (Figure 1C). Excess deaths occurred in 21 of 160 weeks, and the first week with excess deaths in nursing facilities was seen 11 weeks later than in the overall study population. The median percent excesses in 2020, 2021, 2022, and 2023 were −6.0%, 4.6%, 3.1%, and 12.4% respectively.

Regarding deaths at home, no excess deaths were observed until October 2020, and there were fewer-than-expected deaths in 5 weeks (Figure 1D). Excess deaths occurred in 17 of 160 weeks, and the first week with excess deaths at home was seen 11 weeks earlier than in the overall study population. Beginning in 2022, fewer-than-expected deaths were observed in 10 of 56 weeks, while excess deaths were seen in only 2 weeks. The median percent excesses in 2020, 2021, 2022, and 2023 were −1.5%, 8.2%, 5.7%, and −8.3% respectively. Deaths at home were characterized by the largest standard deviation of weekly percent excess when compared to other places. Among deaths at home, the maximum percent excesses of 36.8% and 35.2% were observed in the weeks of December 21–27, 2020, and April 26–May 2, 2021, respectively (eTable 1 and eFigure 2).

## DISCUSSION

This analysis of national data evaluated changing patterns in place of death among patients with dementia during the COVID-19 pandemic. The results indicated that between January and October 2020, the number of people with dementia who died in each place decreased compared with the previous 3 years.

This decrease could be related to a decrease in the incidence of pneumonia around this period. The most common cause of mortality in patients with dementia is pneumonia—the risk of pneumonia-related death in these individuals is almost twice as high as it is in those without dementia, accounting for 29.7% of fatalities.^18^ During the early phase of the pandemic, it was determined that people with advanced age or dementia were at increased risk of COVID-19 infection,^19^^,^^20^ and this may have led care providers to take extra precautions to prevent these populations from being infected with severe acute respiratory syndrome-related coronavirus 2 (SARS-CoV-2). An earlier study of 82 Japanese hospitals showed a 44% to 53% drop in community-acquired pneumonia admissions from April to September 2020 compared to the same period in 2019.^21^

After October 2020, around the time of the third wave, excess deaths were observed among people with dementia in medical institutions, nursing facilities, and homes. During this wave, healthcare institutions reported overwhelming demand related to the care of patients with COVID-19.^22^ People with cognitive decline often cannot report health status changes to a caregiver.^20^ Owing to their problems in understanding, remembering, or acting on public health advice, patients with dementia are more susceptible to SARS-CoV-2 infection and transmission.^23^ It has also been reported that deaths of people with cancer or cerebrovascular disease decreased in medical institutions during the pandemic, while deaths in nursing facilities and at home increased.^24^^,^^25^ Our analysis echoes this previous study in that older adults and family members with diseases such as dementia and cancer tended to choose places other than hospitals as their last place of residence during this period. These factors might have influenced the place of death chosen by these patients and their family members during the pandemic.

Data from 2022 onwards revealed a shift from increased home deaths to an excess of deaths in nursing homes. This period does not correspond to any significant changes in medical policy, but it does coincide with the government’s termination of semi-emergency coronavirus measures,^26^ which can be inferred as marking a shift in Japan’s societal stance towards COVID-19. The prevalence rate of teleworking, which was 31.5% in May 2020, decreased to 18.5% by January 2022,^27^ suggesting changes in the lifestyles of care providers. It can be hypothesized that these societal policies surrounding COVID-19 also impacted the place of death.

In 2017, the Japanese government’s report on advance care planning indicated that more than half the population wished to die in nursing facilities (63.5%, 32.5%, and 3.4% in nursing facilities, homes, and medical institutions, respectively).^28^ A previous population-based study reported an increasing trend in the number of people dying in nursing facilities and homes compared to medical institutions.^11^ Our study demonstrated that in 2021, home death was associated with a higher percent excess (35.2%) than medical institutions and nursing facilities (18.9% and 18.8%, respectively). COVID-19 has forced medical and long-term care institutions to restrict visitation.^29^^,^^30^ In contrast, home visiting care continued to be provided,^31^ enabling patients with dementia and COVID-19 to stay at home until the end of life. Studies have reported bereavement in family members who could not be with loved ones with COVID-19 during their last moments because of visitation restrictions, as well as moral distress in healthcare providers.^32^^–^^34^ Despite the pandemic, person-centered care, including end-of-life care, must be performed. Our study calls for the MHLW to update the national survey and the Survey of Medical Care at the End of Life Stage^28^ to check whether place of death preferences have changed with the pandemic. Additionally, our findings urge physicians to ask patients with dementia and their caregivers about their preferred dying site. This study had some limitations. First, data on the place of death were obtained from death certificates. These may not accurately reflect the preference of the deceased regarding place of death since nursing facility residents with dementia who developed severe COVID-19 might have been transferred to medical institutions and passed away there. Second, in Japan, the rules for selecting the underlying cause of death have been modified since January 2017 to comply with the 2013 edition of ICD-10.^17^ One effect of this rule modification is that in many cases in which the underlying cause of death would previously have been specified as pneumonia, it is now categorized as dementia. Consequently, the number of deaths considered to be due to dementia increased dramatically after January 1, 2017 (eFigure 1), and data used in this study were collected after this modification. Therefore, only the 3-year period of 2017–2019 can be used to estimate the expected number of deaths in 2020. However, for the Farrington algorithm, previous studies have proposed that 3 years of data yield statistically sound results.^35^^,^^36^

In sum, during the early phases of the COVID-19 pandemic in Japan, particularly in 2020, the total number of fatalities among patients with dementia aged 65 years or older was declining. However, excess deaths were observed during and after the third wave transition, which started in October 2020. The 2021 excess of deaths in people with dementia was observed in all places of death, with homes having the highest number of weeks with excess deaths. The findings of this investigation indicate that visitation limits could result in a shift in the desired site of dying. Therefore, in addition to providing the necessary care in this population, physicians may wish to reaffirm their patients’ desired sites of death. These excess deaths also suggest the presence of strained medical resources and limited access to care.

## SUPPLEMENTARY MATERIAL

eMaterial 1. Materials and methods

eMaterial 2. Explanation of abbreviated variable names in eTable 1

eTable 1.

eFigure 1. Weekly trends of the number of deaths considered to be due to dementia between January 2015 and January 2023. The number of deaths considered to be due to dementia increased significantly after January 1, 2017.

eFigure 2. Weekly trends of percent excess of deaths considered to be due to dementia between January 2020 and January 2023.
