# Supplementary material for: Changes in Place of Death Among Patients With Dementia During the COVID-19 Pandemic in Japan: A Time-series Analysis
Source: J Epidemiol. 2024 Oct 5;34(10):493–7. doi: 10.2188/jea.JE20230279 (PMC11405367; doi:10.2188/jea.JE20230279)
Supplement: Supplementary file 1 [file je-34-493-s001.zip › JE20230279_eMaterials_accepted_34-10-clean_EO.pdf]

eMaterial 1. Materials and methods

Study design and population

We used national data from Japan's Ministry of Health, Labour and Welfare (MHLW) between January 2017 and January 2023 for our national-level time series study. We analyzed patients with dementia and examined changes in the numbers of reported deaths and their locations during the coronavirus disease 2019 (COVID-19) pandemic. The National Institute of Infectious Diseases' ethical committee approved this study (authorization no. 1552), and this study adhered to institutional guidelines. Because this was a retrospective, observational study that specifically included de-identified national mortality data, informed consent was waived by the ethics committee. All methods were performed in accordance with relevant guidelines and regulations adhering to the principles of the 1964 Declaration of Helsinki.

Data source

Mortality information was obtained from the MHLW's Vital Statistics. A death certificate in Japan is prepared by a physician within 1 week of the occurrence of death. Place of death, primary cause, and direct cause of death are all listed on the death certificate. All persons with a certificate of residence who die in Japan, regardless of nationality, were included in the mortality data. Those whose place of residence or date of birth is unknown, those who die abroad, and those who stay in Japan for a short period (without a residence card) were not included. Following previous studies, patients aged 65 years or older with vascular dementia (International Classification of Diseases, 10th revision [ICD-10] classification[1] F01), Alzheimer's disease (G30), and dementia not otherwise defined (F03) were included in the analysis.[2–6] The data included counts of deaths where dementia was the direct or indirect cause and included pneumonia deaths related to dementia. There were seven categories of places of death: a) hospitals, b) clinics, c) convalescent hospitals, geriatric healthcare facilities, d) midwifery centers, e) nursing homes, f) homes, and g) others. In the current survey, we re-classified these into four categories based on previous literature: (A) all places, (B) medical institutions (a and b), (C) nursing facilities (c and e), and (D) homes (f).[2–6] As of mid-2022, Japan has faced seven national-level COVID-19 waves, starting at approximately the following time points: April 2020, August 2020, January 2021, May 2021, August 2021, February 2021, August 2022, and January 2023.[7]

Statistical analysis

The estimate of deaths was performed using the quasi-Poisson regression model known as the Farrington algorithm[8, 9] The projected number of fatalities for a certain week, t, was calculated using data from weeks $t – w$ and $t + w$ of years $h – b$ and $h + b$, where w and b are specified parameters and h is the year of t, referred to as the reference period. This method is intended to limit the data utilized for estimation. Thus,

$$\boldsymbol{log}(\boldsymbol{E}(\boldsymbol{Y_t})) = \alpha + \beta \boldsymbol{t} + \boldsymbol{f^T}(\boldsymbol{t})\boldsymbol{\gamma_{f(t)}}$$

can be used to define the quasi-Poisson regression analysis utilized in the Farrington algorithm, where Y_t is the number of deaths in a given week and is assumed to follow a quasi-Poisson distribution with a dispersion value. Regression parameters α and β, a vector of dummy variables called $\boldsymbol{f}(\boldsymbol{t})$ that evenly split time points outside the reference period, and a regression parameter vector $\boldsymbol{\gamma_{f(t)}}$ that represents seasonality were all included in the analysis. To regulate seasonality, the study separated data for a 1-year period that was not part of the reference period into nine periods, following prior studies.[10, 11]

In Japan, the criteria for determining the primary cause of death have changed since January 2017 to conform to the partial revision of the ICD-10 in 2013.[12] To avoid inconsistencies, therefore, data before 2017 were not used. Using data for 3 weeks before and after each week (w=3) in the past 3 years (b=3) for 21 weeks, the expected number of deaths for a given week during the COVID-19 pandemic was calculated, assuming that the number of deaths would follow pre-pandemic historical trends. The reason for using data from 3 years was the change in criteria in 2017, as mentioned above. The estimates were calculated using 95% two-sided prediction intervals for the upper and lower limits. The discrepancy between the actual and expected death toll was used to compute the number of excess deaths. We defined percent excess as a relative measure of the magnitude of the excess. The formula for the percent excess is ((observed-expected)/expected)*100. Based on the category of the place of deaths specified in the National Institute of Infectious Diseases' Infectious Diseases Weekly Report, daily data were converted to weekly data.[13] For all analyses and graphical output, we used R version 4.1.0 (R Foundation for Statistical Computing, Vienna, Austria). The Farrington algorithm was analyzed by the R package “surveillance.”[14] The week count

used in the study aligns with the epidemiological week defined by the National Institute of Infectious Diseases.

Since we only used pre-pandemic data from 2017 to 2019 (see limitations in the Discussion), we set the algorithm to use up to the last 3 years of data to predict the expected number of deaths in a given week. Under this setup, if only pre-pandemic data were used for estimation during the pandemic, data from the 3 years prior to the pandemic (2017–2019) could be used for estimation in 2020. However, this would mean that only 2 years of data for the pre-pandemic period, 2018–2019, could be used for the 2021 estimate. Due to the insufficient stability of the model with less than 2 years of data, we utilized Anscombe residuals to weight outliers (such as excesses) during the pandemic, enabling the use of data from 2020 onwards for the estimates for 2021–2023.[8] In our algorithm, the Anscombe residual, S_i, for a given week t_i was used to determine the weighting. If S_i was greater than 1, a lower weight was assigned by multiplying the existing weight by S_i^{-2}. If S_i was less than or equal to 1, the weight remained unchanged. This method ensured that weeks with typical counts, as indicated by lower residuals, were given more weight in the analysis. Conversely, atypical weeks with standout counts, which were represented by higher residuals, were given less weight, thereby diminishing their impact on the estimation.

REFERENCES

1. World Health Organization. The ICD-10 Classification of Mental and Behavioural Disorders: Clinical Descriptions and Diagnostic Guidelines. Geneva: World Health Organization; 1992.
2. Koyama T, Sasaki M, Hagiya H, et al. Place of death trends among patients with dementia in Japan: a population-based observational study. Sci Rep. 2019;9(1):20235. doi: 10.1038/s41598-019-56388-w.
3. Koyama T, Hagiya H, Funahashi T, et al. Trends in place of death in a super-aged society: a population-based study, 1998-2017. J Palliat Med. 2020;23(7):950-956. doi: 10.1089/jpm.2019.0445.
4. Sleeman KE, Ho YK, Verne J, Gao W, Higginson IJ, GUIDE_Care project. Reversal of English trend towards hospital death in dementia: a population-based study of place of death and associated individual and regional factors, 2001-2010. BMC Neurol. 2014;14:59. doi: 10.1186/1471-2377-14-59.
5. Houttekier D, Cohen J, Bilsen J, Addington-Hall J, Onwuteaka-Philipsen BD, Deliens L. Place of death of older persons with dementia. A study in five European countries. J Am Geriatr Soc. 2010;58(4):751-756. doi: 10.1111/j.1532-5415.2010.02771.x.
6. Mitchell SL, Teno JM, Miller SC, Mor V. A national study of the location of death for older persons with dementia. J Am Geriatr Soc. 2005;53(2):299-305. doi: 10.1111/j.1532-5415.2005.53118.x.
7. Ministry of Health, Labour and Welfare. Visualizing the data: information on COVID-19 infections (2022). https://covid19.mhlw.go.jp/extensions/public/en/index.html [Accessed July 23, 2023].
8. Noufaily A, Enki DG, Farrington P, Garthwaite P, Andrews N, Charlett A. An improved algorithm for outbreak detection in multiple surveillance systems. Stat Med. 2013;32(7):1206-1222. doi: 10.1002/sim.5595.
9. Farrington CP, Andrews NJ, Beale AD, Catchpole MA. A statistical algorithm for the early detection of outbreaks of infectious disease. J R Stat Soc A. 1996;159(3):547-563. doi: 10.2307/2983331.
10. Nomura S, Eguchi A, Ghaznavi C, et al. Excess deaths from non-COVID-19-related causes in Japan and 47 prefectures from January 2020 through May 2021

by place of death. SSM Popul Health. 2022;19:101196. doi: 10.1016/j.ssmph.2022.101196.

11. Centers for Disease Control and Prevention. Excess deaths associated with COVID-19. https://www.cdc.gov/nchs/nvss/vsrr/covid19/excess_deaths.htm Accessed March 30 2023; 2022.
12. Ministry of Health, Labour and Welfare. The impact of partial application of the tenth revision of the international statistical classification of diseases and related health problems (ICD-10) on cause of death statistics. https://www.mhlw.go.jp/toukei/list/dl/icd_2013_eikyo.pdf Accessed March 30 2023; 2018.
13. National Institute of Infectious Diseases. Report Week Correspondence Table [Japanese]. https://www.niid.go.jp/niid/ja/calendar.html Accessed March 30 2023; 2021.
14. Salmon M, Schumacher D, Höhle M. Monitoring Count Time Series in R: Aberration Detection in Public Health Surveillance. J Stat Softw. 2016;70(10). doi: 10.18637/jss.v070.i10.

eMaterial 2. Explanation of abbreviated variable names in eTable 1

All_observed (n)	The number of all observed deaths
All_expected (n)	The number of all expected deaths
All_95u (n)	The number of 95% upper bound of all expected deaths
All_95l (n)	The number of 95% lower bound of all expected deaths
All_excess (n)	The number of excesses observed deaths from all expected deaths
All_percent (%)	The percent of excesses observed deaths from all expected deaths
MedicalFacilities_observed (n)	The number of observed deaths in medical facilities
MedicalFacilities_expected (n)	The number of expected deaths in medical facilities
MedicalFacilities_95u (n)	The number of 95% upper bound of expected deaths in medical facilities
MedicalFacilities_95l (n)	The number of 95% lower bound of expected deaths in medical facilities
MedicalFacilities_excess (n)	The number of excesses observed deaths from expected deaths in medical facilities
MedicalFacilities_percent (%)	The percent of excesses observed deaths from expected deaths in medical facilities
Nursing_observed (n)	The number of observed deaths in nursing homes
Nursing_expected (n)	The number of expected deaths in nursing homes
Nursing_95u (n)	The number of 95% upper bound of expected deaths in nursing homes
Nursing_95l (n)	The number of 95% lower bound of expected deaths in nursing homes
Nursing_excess (n)	The number of excesses observed deaths from expected deaths in nursing homes
Nursing_percent (%)	The percent of excesses observed deaths from expected deaths in nursing homes
Home_observed (n)	The number of observed deaths in homes
Home_expected (n)	The number of expected deaths in homes
Home_95u (n)	The number of 95% upper bound of expected deaths in homes
Home_95l (n)	The number of 95% lower bound of expected deaths in homes
Home_excess (n)	The number of excesses observed deaths from expected deaths in homes
Home_percent (%)	The percent of excesses observed deaths from expected deaths in homes

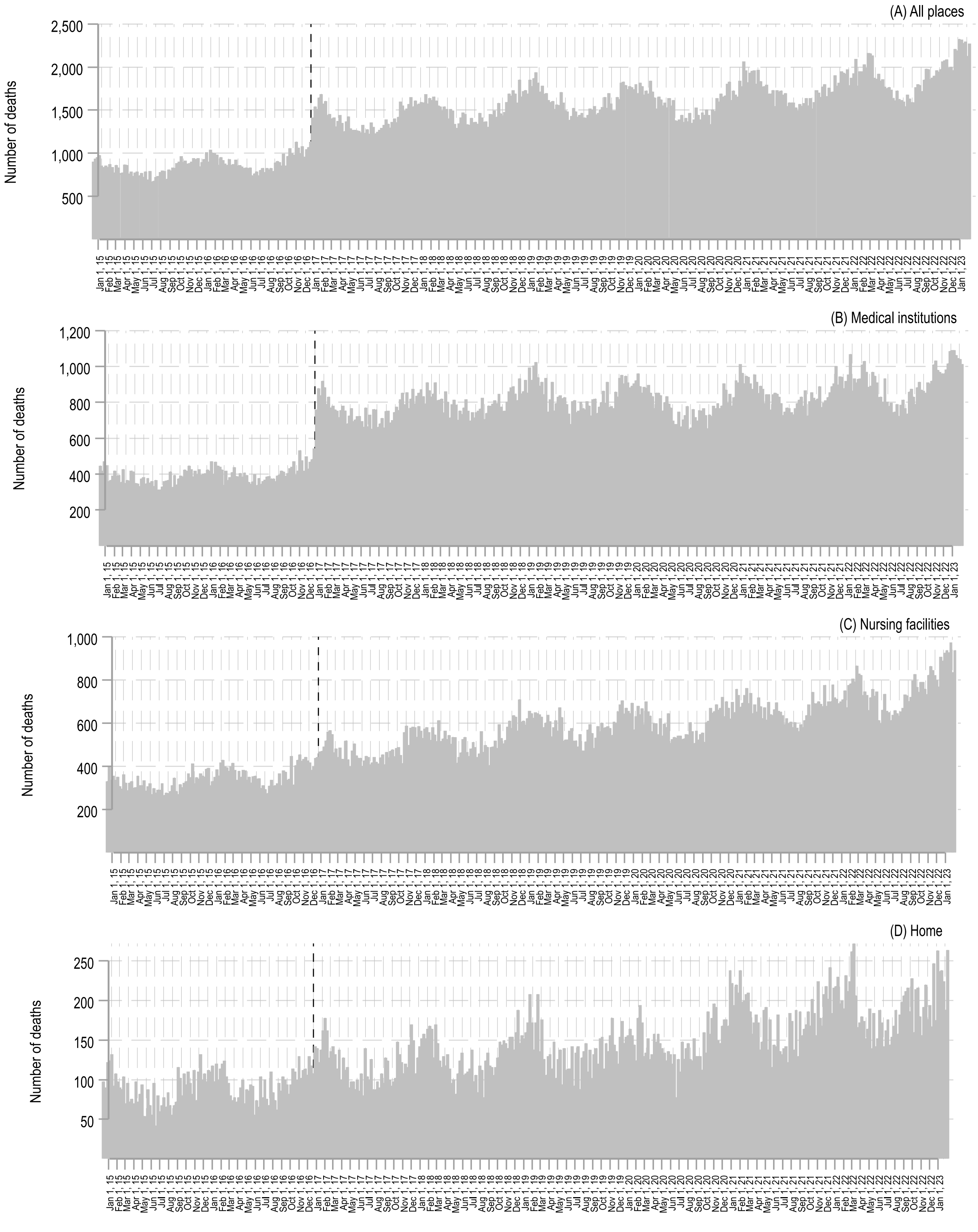

eFigure 1. Weekly trends of the number of deaths considered to be due to dementia between January 2015 and January 2023. The number of deaths considered to be due to dementia increased significantly after January 1, 2017.

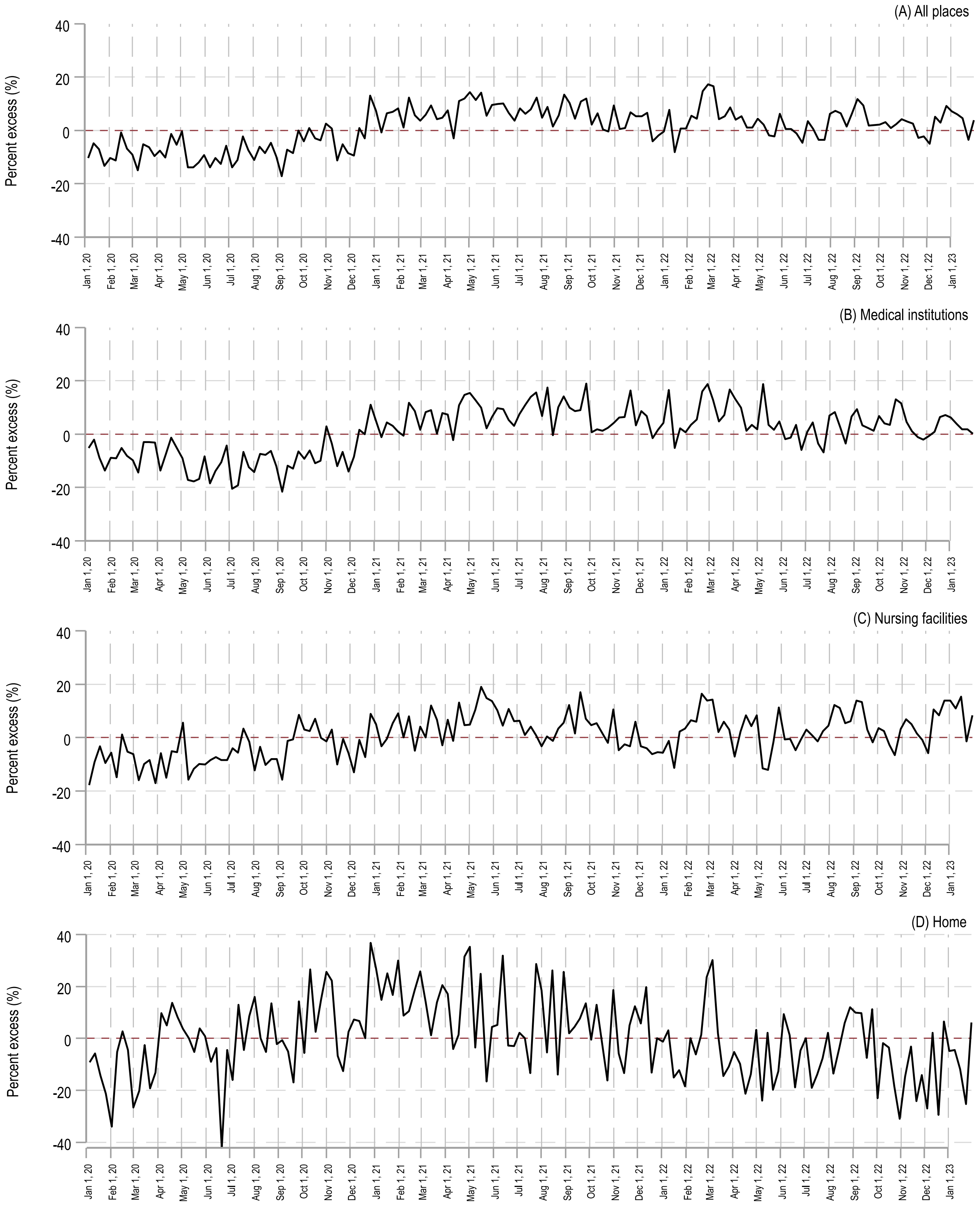

eFigure 2. Weekly trends of percent excess of deaths considered to be due to dementia between January 2020 and January 2023.
